# Molecular pathway identification using biological network-regularized logistic models

**DOI:** 10.1186/1471-2164-14-S8-S7

**Published:** 2013-12-09

**Authors:** Wen Zhang, Ying-wooi Wan, Genevera I Allen, Kaifang Pang, Matthew L Anderson, Zhandong Liu

**Affiliations:** 1 Department of Pediatrics-Neurology, Baylor College of Medicine, Houston, TX, USA; 2 Jan and Dan Duncan Neurological Research Institute at Texas Children's Hospital, Houston, TX, USA; 3 Department of Obstetrics and Gynecology, Baylor College of Medicine, Houston, TX, USA; 4 Department of Statistics and Electrical Engineering, Rice University, Houston, TX, USA; 5 Computational and Integrative Biomedical Research Center, Baylor College of Medicine, Houston, TX, USA; 6 Dan L. Duncan Cancer Center, Baylor College of Medicine, Houston, TX, USA

## Abstract

**Background:**

Selecting genes and pathways indicative of disease is a central problem in computational biology. This problem is especially challenging when parsing multi-dimensional genomic data. A number of tools, such as *L*_1_-norm based regularization and its extensions elastic net and fused lasso, have been introduced to deal with this challenge. However, these approaches tend to ignore the vast amount of *a priori *biological network information curated in the literature.

**Results:**

We propose the use of graph Laplacian regularized logistic regression to integrate biological networks into disease classification and pathway association problems. Simulation studies demonstrate that the performance of the proposed algorithm is superior to elastic net and lasso analyses. Utility of this algorithm is also validated by its ability to reliably differentiate breast cancer subtypes using a large breast cancer dataset recently generated by the Cancer Genome Atlas (TCGA) consortium. Many of the protein-protein interaction modules identified by our approach are further supported by evidence published in the literature. Source code of the proposed algorithm is freely available at http://www.github.com/zhandong/Logit-Lapnet.

**Conclusion:**

Logistic regression with graph Laplacian regularization is an effective algorithm for identifying key pathways and modules associated with disease subtypes. With the rapid expansion of our knowledge of biological regulatory networks, this approach will become more accurate and increasingly useful for mining transcriptomic, epi-genomic, and other types of genome wide association studies.

## Introduction

Technologies for high throughput genetic profiling have revolutionized the study of human development and disease. Expression profiles spanning the entire human genome not only allow investigators to better understand disease subtypes [1], but also define new categories associated with sensitivity to pharmacologic treatment [2,3] and other clinical outcomes [4,5]. A central problem in these genomic studies is to construct an accurate predictive model and delineate specific genes or pathways driving a phenotype. Logistic regression is widely used for classification [6-9]. The number of genes, however, in high-throughput studies are often much larger than the number of specimens in a given study. This limitation causes instability in the algorithms used to select driver genes and poor performance of predictive models. The lasso algorithm for logistic regression was introduced to address these problems and perform feature selection through *L*_1_-norm regularization [10]. However, the number of variables selected by the lasso is bounded by the number of observations in an experiment. Furthermore, correlated variables are rarely selected as part of the predictive model at the same time. Various extensions of the Lasso algorithm, such as elastic net, pelora, grouped lasso and fused lasso introduce grouping or smoothness regularization terms to address these limitations [11-14]. Of these, the fused lasso and elastic net have been successfully applied to a largest number of gene expression and genome wide association studies [15-18]. Both fused Lasso and elastic net allow correlated genes or neighboring genes on a chromosome to be selected into a predictive model together. However, these algorithms tend to ignore functional interactions between individual gene products documented in the scientific literature. Integration of network information in the gene marker identification has shown to outperform methods without network information by Chuang et al [19]. Specifically, their networked-based scoring and greedy search algorithm identified more robust gene markers with better prediction accuracy on metastasis status of breast cancer patients in two cohorts. In recent years, vast amounts of data detailing biologic networks has been organized into searchable databases. For example, BioGRID documents protein and genetic interactions from more than 39,991 publications [20]. The KEGG pathway database similarly curates molecular interactions and relational networks representing systemic functions at the level of both the cell and organism [21]. To incorporate biological network information into regression models, a network-constrained regularization algorithm has been previously proposed for use with linear regression [22]. Use of this network-constrained algorithm has been shown to out-perform both lasso and elastic net analyses executed independently of biologic input.

Classification algorithms integrating network structure information have been proposed in other settings. For example, a network constrained support vector machine has been proposed to analyze functional magnetic resonance imaging data [23] and cancer microarray data [24-26]. Network based prior has also been demonstrated to improve variable selection accuracy under the Bayesian inference framework [27,28].

Here, we propose a graphical Laplacian network regularized logistic regression method following the framework established by Li et al. [22]. We hypothesize that the integration of biological networks, such as Protein-Protein interactions, will improve prediction accuracy and variable selection in logistic models. To validate use of the proposed algorithm, we studied its theoretical properties and compared its performance to *L*_1_-norm regularized logistic regression and elastic net logistic regression on both simulated and real biologic data. Lastly, we demonstrate the utility of the proposed algorithm by using it to differentiate breast cancer subtypes and delineate biologic network modules associated with the triple negative breast cancer (TNBC) subtypes.

## Materials and methods

### Graph laplacian regularized logistic regression model

Suppose that the data set has *n *observations with *p *genes. Let **y **= (y_1_, ..., y*_n_*)^*T *^be the response with y*_i _*∈ {0, 1} and **X **= [**x_1_**|, ..., |**x_p_**] be the matrix of biomarkers measured on *n *samples with **x_j _**= (*x*_1*j*_, ..., *x_nj_*)^*T *^for *j *= 1, ..., *p *genes. Without loss of generality, we can assume that each gene is standardized. The binary clinical variable, **y**, can be predicted using the logistic model:

(1) $$\text{Prob}\;\;\;\left(y=1|\text{X};\;\beta \right)=\frac{1}{1+e^{-\text{X}\beta }}$$

The parameter *β *can be estimated by maximizing the log likelihood function of the logistic model. However, it is well known that this estimation procedure performs poorly for both prediction purposes and variable selection when *p *≫ *n*. Although various sparse parameter estimation procedures have been introduced to address these problems, these approaches tend to generate disconnected biomarkers that are rarely interpretable.

To incorporate biological network information into the model estimation procedure, we adopted the network-constrained regularization framework proposed by Li et al [22]. Given a biological network *G *= (*V*, *E*), where *V *is the set of genes that correspond to *p *predictors, *E *is the adjacency matrix, and *e_uv _*= 1 if there exists an edge between *u *and *v*, otherwise *e_uv _*= 0. The normalized graph Laplacian matrix *L *for *G *can be defined by

(2) $$L=\text{I}-D^{-\frac{1}{2}}ED^{-\frac{1}{2}}$$

where *D *= diag(*E*·1*_p_*) is a degree matrix with the diagonal elements equal to the degrees for each node in *G*. For any fixed non-negative regularization parameters *λ *and *α*, we define the logistic graph Laplacian net (Logit-Lapnet) criteria as:

(3) $$L\left(\lambda ,\alpha ,\beta \right)=\overset{n}{\underset{i=1}{\sum }}\left[-y_{i}X_{i}\beta +\ln\left(1+e^{X_{i}\beta }\right)\right]\;+\;\lambda \alpha |\beta {|}_{1}+\lambda \left(1-\alpha \right){\left\langle \beta ,\beta \right\rangle }_{L}$$

Where

$$\begin{array}{c} |\beta {|}_{1}=\overset{p}{\underset{j=1}{\sum }}|\beta_{j}|,\\ {\left\langle \beta ,\beta \right\rangle }_{L}=\beta^{T}L\beta =\underset{e_{uv}\neq 0}{\sum }{\left(\frac{\beta_{u}}{d_{u}}-\frac{\beta_{v}}{d_{u}}\right)}^{2}. \end{array}$$

*d_u_*, *d_v _*are the degrees of nodes *u *and *v *respectively. In (3), *X_i _*is the *i*th row of the matrix *X*. The first term in equation (3) is the negative log likelihood function of the logistic model. The second term is an *L*_1_-norm penalization on *β*, which encourages sparsity on the coefficients. The last term is a generalized *L*_2_-norm penalty using the graph Laplacian matrix, which encourages smoothness on coefficients of genes that are connected in the biological network. If we set *α *= 1, the Logit-Lapnet criteria is equal to the simple lasso logistic regression. When *L *= **I **which is identity matrix, the Logit-Lapnet criteria becomes the elastic net logistic regression.

The model estimation can be formulated into a convex optimization problem:

(4) $$\hat{\beta }=\arg\underset{\beta }{\min}L\left(\lambda ,\alpha ,\beta \right).$$

To solve problem (4) we used CVX, a package for specifying and solving convex programs [29,30]. The convexity property of Logit-Lapnet guarantees an optimal solution using any convex optimization solver.

### Theoretical properties of logit-lapnet

To study the behavior of the proposed method, we analyzed the theoretical properties of Logit-Lapnet.

**Lemma 1 ***If *$x_{i}=x_{j}$, *then *${\hat{\beta }}_{i}={\hat{\beta }}_{j}$*for any α *< 1 *and λ *> 0.

The proof of this lemma is given in the additional file 1. This lemma states that if two predictors are identical, the coefficients of these two predictors will be the same. In the *L*_1_-norm penalized logistic regression, only one predictor is selected.

**Theorem 1 ***Given data *(**y**, **X**) *and parameters *(*λ*, *α*), *let *$\hat{\beta }$(*λ*, *α*) *be the Logit-Lapnet estimator for problem (4). If ${\hat{\beta }}_{i}{\hat{\beta }}_{j}>0\;$ and e_ij _= *1, *define *$\;D_{\lambda ,\alpha }\left(i,j\right)=\frac{1}{|\text{y}{|}_{1}}|{\hat{\beta }}_{i}\left(\lambda ,\alpha \right)-{\hat{\beta }}_{j}\left(\lambda ,\alpha \right)|$*, then*

(5) $$D_{\lambda ,\alpha }(i,j)\leq \frac{\sqrt{2(1-\rho })}{2\lambda (1-\alpha )}$$

*where ρ  is the sample correlation of ***x***_i _and ***x***_j _*.

The upper bound in (5) provides a quantitative description for the grouping effect of Logit-Lapnet on the network structure. For two highly correlated genes (ρ=1) that are connected in a biological network, the difference on the estimated coefficient is almost zero. As *α *goes to 1, the Lapnet becomes lasso logistic regression and the difference becomes unbounded.

**Lemma 2 ***The maximum value of λ in problem (4) with *$\hat{\beta }\neq 0$*satisfies*

(6) $$\lambda_{\max}\leq \frac{1}{2\alpha }\left(2|{\text{X}}^{T}\text{y}{|}_{\infty }+|\overset{n}{\underset{i=1}{\sum }}X_{i}^{T}{|}_{\infty }\right)$$

The maximum value of *λ *is reciprocal to *α*. It is clear that when *α *= 1, the Logit-Lapnet criteria becomes Lasso and a small penalization can produce an empty model. When *α *= 0, the penalization becomes much larger to generate an empty model. In practice, Lemma 2 provides a search guidance on the regularization path. The proof of Lemma 2 is also given in the additional file 1.

### Gene expression profiles and differential gene analysis

Gene expression data from breast cancer specimens profiled by TCGA consortium was used to test the proposed Logit-Lapnet method. Level III RNA-Seq data (Illumina HigSeq RNASeqV2 from UNC) from patients with invasive breast carcinoma was obtained from the TCGA data portal https://tcga-data.nci.nih.gov/tcga/ in September, 2012. These data profiled 20501 genes in 806 distinct breast cancer specimens. Normalized read counts were used for all analyses and were log-transformed prior to their use. Genes with normalized read counts less than 10 in more than 90% of patients were excluded from further analyses. The normalized read counts were log-transformed and standardized prior to applying the method.

Of the 806 breast cancers characterized by the TCGA, 261 patients had incomplete information on the three markers used to define TNBC and were excluded. Of the remaining cancers, 85 TNBC and 460 non-TNBC were further separated into training (n = 327; 51 TNBC, 276 non-TNBC) and test (n = 218; 34 TNBC, 184 non-TNBC) sets. A total of 4871 differentially expressed gene products (P ≤ 0.05, t-test; ≥ 1.5 fold-change between TNBC vs. non-TNBC on training) were merged with protein-protein interaction (PPI) networks. This provided us with 797 genes and PPI networks of 937 interactions available for analysis using our proposed algorithm.

### Protein-protein interaction (PPI) network

To construct the graph Laplacian matrix for testing the Logit-Lapnet method on breast cancer data, networks of protein-protein interactions (PPI) identified by two-hybrid screening in Homo sapiens were obtained from The Biological General Repository for Interaction Datasets (BioGRID, version 3.2.98) http://www.thebiogrid.org. At the time it was accessed, BioGRID documented 21483 interactions between 7700 genes.

## Results and discussion

### Simulation studies

We initially used a benchmark simulation proposed by Li to explore the performance of the proposed Logit-Lapnet algorithm [22]. In brief, a network with 200 distinct transcription factors (TFs) was simulated. Each TF in this simulation regulated 10 genes with a total of 2,200 genes in the simulated network. The clinical variable y was assigned a binary value and was associated with the first four TFs and their target genes. In model I, we assumed that two of the TFs and their targets were positively associated with the clinical variable and the other two TFs and their targets were negatively associated with the clinical variable.

$$\beta ={\left(5,\underset{10}{\underset{︸}{\frac{5}{\sqrt{10}}\;,\;.\;.\;.\;,\;\frac{5}{\sqrt{10}},}}-5,\underset{10}{\underset{︸}{\frac{-5}{\sqrt{10}},\;.\;.\;.\;,\;\frac{-5}{\sqrt{10}},}}3,\underset{10}{\underset{︸}{\frac{3}{\sqrt{10}}\;,\;.\;.\;.\;,\frac{3}{\sqrt{10}},}}-3,\underset{10}{\underset{︸}{\frac{-3}{\sqrt{10}},...,\frac{-3}{\sqrt{10}}\;,\;}}0,\;.\;.\;.\;,\;0\right)}^{T}$$

The clinical variable was defined as y = [Prob (y = 1|X; *β*) *> ϱ*] where *ϱ *~ *U*(0,1). Expression levels for the 200 TFs were then simulated using a standard normal distribution. Each TF and its target genes were jointly distributed as a bivariate normal with correlation of 0.7.

In model II, gene expression levels were simulated similarly to model I except that a TF could be both a transcriptional activator and repressor at the same time. The coefcient vector was defined as:

$${\beta =(5,\frac{-5}{\sqrt{10}},\frac{-5}{\sqrt{10}},\frac{-5}{\sqrt{10}},\underset{7}{\underset{︸}{\frac{5}{\sqrt{10}},\;.\;.\;.\;,\frac{5}{\sqrt{10}}}},-5,\;\frac{5}{\sqrt{10}},\frac{5}{\sqrt{10}},\frac{5}{\sqrt{10}},\underset{7}{\underset{︸}{\frac{-5}{\sqrt{10}}\;,\;.\;.\;.\;,\;\frac{-5}{\sqrt{10}}}},3,\frac{-3}{\sqrt{10}},\frac{-3}{\sqrt{10}},\frac{-3}{\sqrt{10}},\underset{7}{\underset{︸}{\frac{3}{\sqrt{10}}\;,\;.\;.\;.\;,\;\frac{3}{\sqrt{10}}}},-3,\frac{3}{\sqrt{10}},\frac{3}{\sqrt{10}},\frac{3}{\sqrt{10}},\underset{7}{\underset{︸}{\frac{-3}{\sqrt{10}}\;,\;.\;.\;.\;,\frac{-3}{\sqrt{10}}}},0\;,\;.\;.\;.\;,\;0)}^{T}$$

Model III was similar to model I except that we decreased the association of the target genes on the clinical variable and made the model even sparser.

$$\beta ={\left(5,\underset{10}{\underset{︸}{\frac{5}{10}\;,\;.\;.\;.\;,\;\frac{5}{10},}}-5,\underset{10}{\underset{︸}{-\frac{5}{10}\;,\;.\;.\;.\;,\;-\frac{5}{10},}}3,\underset{10}{\underset{︸}{\frac{3}{10}\;,\;.\;.\;.\;,\;\frac{3}{10},}}-3,\underset{10}{\underset{︸}{-\frac{3}{10}\;,\;.\;.\;.\;,\;-\frac{3}{10},}}0\;,\;.\;.\;.\;,\;0\right)}^{T}$$

Model IV was similar to model II in allowing transcription factors to function as both activators and repressors. However, the clinical association of the target genes was decreased.

$${\beta =(5,-\frac{5}{10},-\frac{5}{10},-\frac{5}{10},\underset{7}{\underset{︸}{\frac{5}{10}\;,\;.\;.\;.\;,\;\frac{5}{10}}},-5,\frac{5}{10},\frac{5}{10},\frac{5}{10},\underset{7}{\underset{︸}{-\frac{5}{10}\;,\;.\;.\;.\;,\;-\frac{5}{10}}},3,-\frac{3}{10},-\frac{3}{10},-\frac{3}{10},\underset{7}{\underset{︸}{\frac{3}{10}\;,\;.\;.\;.\;,\;\frac{3}{10}}},-3,\frac{3}{10},\frac{3}{10},\frac{3}{10},-\underset{7}{\underset{︸}{\frac{3}{10}\;,\;.\;.\;.\;,\;-\frac{3}{10}}},0\;,\;.\;.\;.\;,\;0)}^{T}$$

For each model, we simulated both a training data set as well as an independent test data set of 100 samples. A 10-fold cross-validation procedure was applied to the training data set to identify the optimal tuning parameter. Genes with non-zero coefficient in the estimated model were found to be associated with the clinical variable. The sensitivity and specificity of our variable selection performance was defined as the following:

(7) $$\begin{array}{c} \text{True Negative (TN)}:=\;|\bar{\beta }.*\bar{\hat{\beta }}{|}_{0},\;\text{False Positive (FP)}\;\;\text{: = }\;|\bar{\beta }\text{.}*\hat{\beta }{|}_{\text{0}}\\ \text{False Negative (FN)}:=\;|\beta .*\bar{\hat{\beta }}{|}_{0},\;\text{True Positive (TP)}\;\;\text{: = }\;|\beta \text{.}*\hat{\beta }{|}_{\text{0}}\\ \;\text{Sensitivity}\;\;\text{: = }\frac{\text{TP}}{\text{TP}+\text{FN}},\;\text{Specificity}\;\;\text{: = }\frac{\text{TN}}{\text{TN}+\text{FP}} \end{array}$$

where the |·|_0 _counts the number of non-zero elements in a vector, $\bar{\beta }$ is the logical not operator on a vector and.* is the element-wise product.

**Figure 1:**
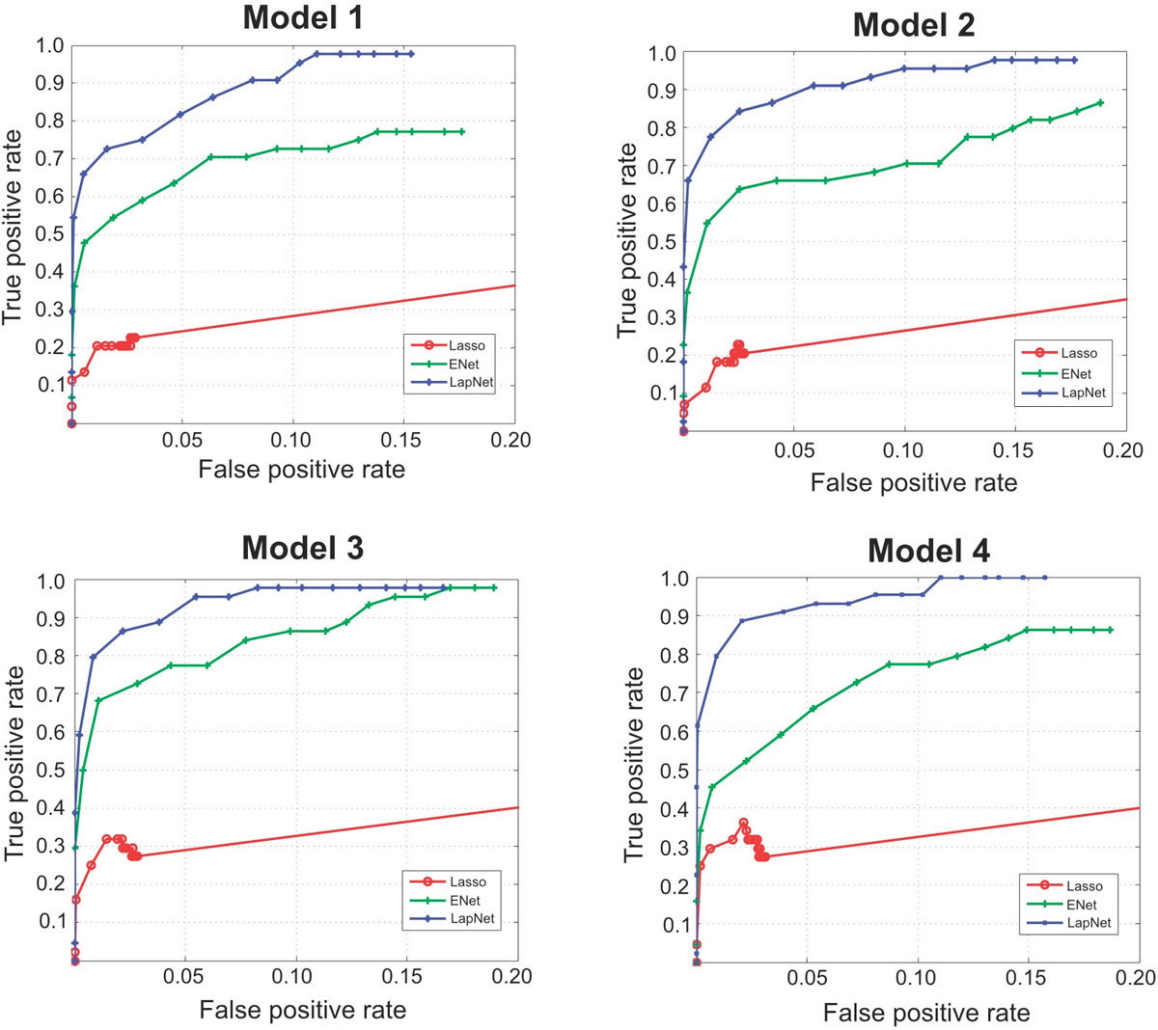
**Receiver operator curves (ROCs) on the regularization path**. ROCs were computed on the regularization path for Logit-Lapnet, enet, and lasso for all four models (A)-(D). Logit-Lapnet has higher true positive rate and lower false positive rate compared to the other two approaches.

We repeated the experiment 50 times. Results are summarized for each model in Table 1. We also computed the Bernoulli error loss on the test data set. For all four models, we compared the performance of the proposed algorithm to both the *L*_1 _penalized logistic regression and the elastic net algorithm. Our method resulted in much higher sensitivity in identifying associated genes with the same specificity compared to the other two algorithms (Table 1). Our method also gave much smaller MSE compared to the Lasso and elastic net logistic regression. We computed the Receiver Operator Curve on the whole regularization path for each of the algorithm. In all four models, the proposed algorithm demonstrated much higher precision compared to Lasso and elastic net logistic regression (Figure 1).

**Table 1:** Results of simulations.

	Sensitivity			Specificity			Bernoulli Error Loss		
**#**	**Lasso**	**Elastic**	**Lapnet**	**Lasso**	**Elastic**	**Lapnet**	**Lasso**	**Elastic**	**Lapnet**

1	0.3146	0.4995	**0.7583 **	0.9744	**0.9945 **	0.9832	19.24	17.2	**13.84 **
	(0.059)	(0.069)	(0.071)	(0.0028)	(0.002)	(0.005)	(0.578)	(0.537)	(0.493)

2	0.1852	0.4386	**0.6577 **	**0.9982 **	0.9936	0.9847	19.22	19.62	**16.8 **
	(0.042)	(0.076)	(0.079)	(0.001)	(0.003)	(0.005)	(0.702)	(0.671)	(0.571)

3	0.2614	0.5714	**0.8832 **	0.9887	**0.9893 **	0.9537	15.14	16.44	**14.38 **
	(0.045)	(0.068)	(0.066)	(0.0024)	(0.0026)	(0.006)	(0.582)	(0.585)	(0.495)

4	0.2314	0.6755	**0.8645 **	**0.9927 **	0.9583	0.9492	17.38	18.86	**16.1 **
	(0.043)	(0.0712)	(0.069)	(0.0019)	(0.0045)	(0.009)	(0.511)	(0.572)	(0.570)

The results for simulations. Sensitivity, specificity and PMSEs are based on 50 simulations. The standard errors are given in parentheses.

### Biomarker identification using logit-lapnet in human breast cancers

Breast cancers are clinically categorized according to the expression of several gene products, including the estrogen receptor (ESR1), progesterone receptor (PGR) and human epidermal growth factor receptor 2 (ERBB2). These biomarkers are routinely used not only to define prognosis but also determine treatment. [31,32]. Of the breast cancer subtypes defined by these biomarkers, triple negative breast cancers (TNBC) lacking expression of ESR1, PAR and ERBB2(her2) are the most clinically aggressive. TNBC demonstrate high rates of disease progression and recurrence [33]. Outcomes for this breast cancer subtype are generally poor, largely due to the fact that treatment options for women with TNBC are limited. However, a subgroup of TNBC are highly sensitive to conventional chemotherapy [34].

We chose to next validate use of our method by testing its ability to identify the key biomarkers used to define breast cancer subtypes. To accomplish this goal, we used patterns of gene expression in a large set of breast cancer specimens recently profiled by the Cancer Genome Atlas consortium. The tuning parameter for each method was selected through 10-fold cross-validation using the training data. We observed the same classification accuracy in predicting TNBC tumors (95%) when lasso, elastic net and Logit-Lapnet were applied to the testing data. The similar efficacy of these 3 algorithms is not surprising since TNBC tumors are dramatically different from the non-TNBCs in terms of their gene expression profiles.

**Figure 2:**
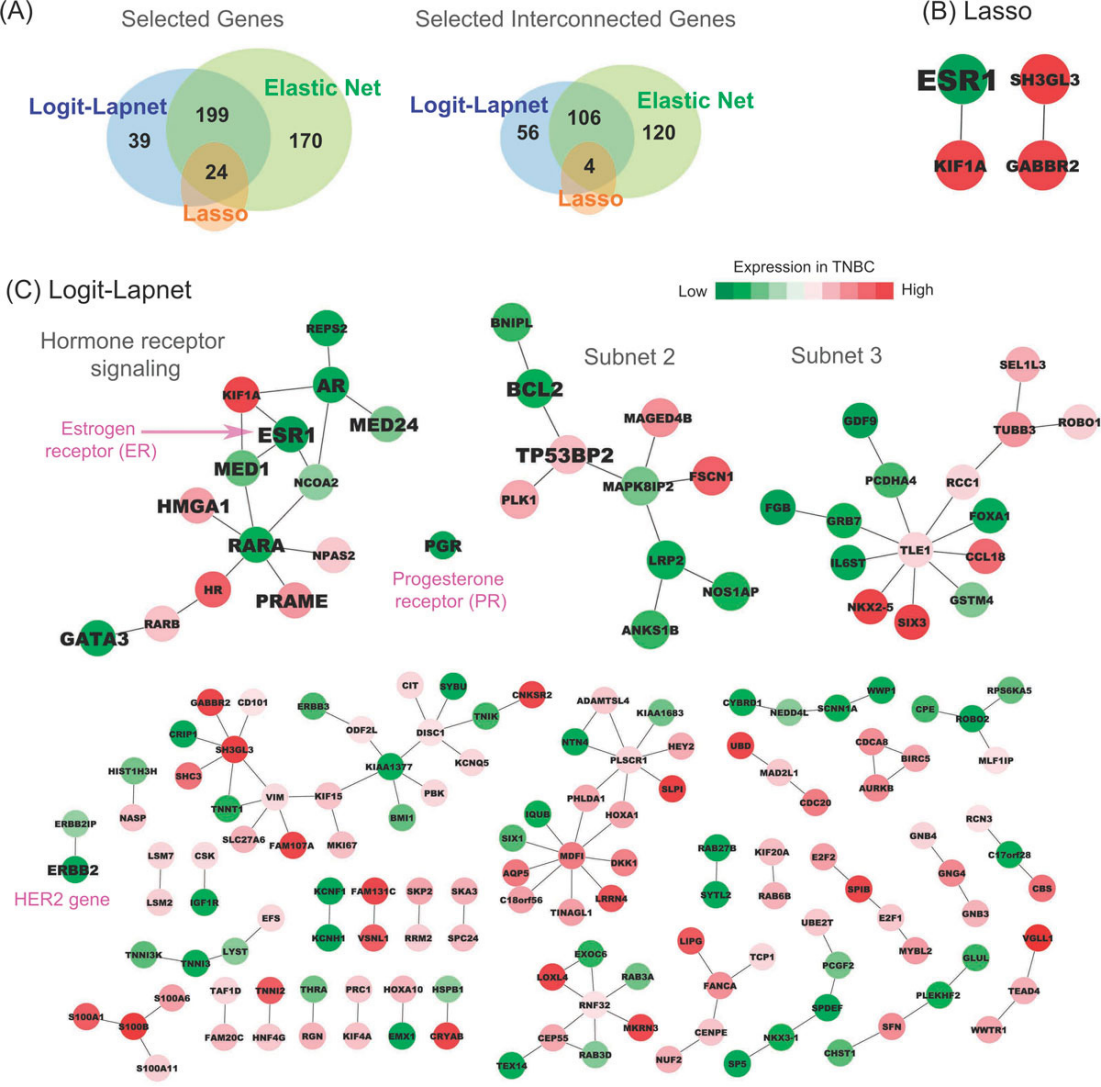
**Application of the algorithm to identify TNBC-associated genes**. Genes and subnetworks of PPI associated with TNBC using TCGA breast cancer data and BioGRID PPI. Comparison of the selected genes from our proposed algorithm with those from lasso and elastic net (A). Genes and their respective subnetworks of PPI recovered by lasso (B) and our proposed Laplacian net algorithm (C). In the networks, genes having association to breast cancer reported in the literature are labeled with larger font.

However, there were a number of significant differences observed between the results obtained with the 3 algorithms tested. For example, use of Logit-Lapnet selected 262 genes, of which > 63% (166 genes) interacted with one another (Figure 2A). In comparison, use of the Lasso algorithm selected only 24 genes, most of which (20 genes) were isolated and were not predicted to interact. Elastic net selected nearly half of the input genes (393 genes) of which 59% (230 genes) were interconnected (Additional File 2). Furthermore, neither Lasso nor elastic net identified the progesterone receptor as a key discriminator for TNBC, despite the fact that this gene product is routinely used to clinically categorize breast cancers. Only Logit-Lapnet successfully identified each of the three markers used to define TNBC subtype: ESR1, PGR and ERBB2. These results suggest that Logit-Lapnet is more accurate than either Lasso or elastic net for identifying biomarkers from large multidimensional genomic datasets such as those generated by the TCGA.

### Use of logit-lapnet for inferring novel biologic relationships

In addition to identifying each of the 3 key biomarkers used to categorize breast cancer subtypes, we found that our method identified multiple subnetworks of gene expression in the breast cancer specimens profiled by TCGA. These subnetworks potentially reflect relationships with clinical or biologic significance. For example, one of subnetworks we identified includes multiple genes (AR, ESR1, MED1, MED24, RARA, PRAME, and HMGA1) involved in steroid hormone signaling. As demonstrated in Figure 2, Logit-Lapnet found that this cluster is closely connected to a known breast cancer gene, GATA3. This connection suggests a functional relationship. Evidence to support this relationship could not be found by directly searching the database of protein-protein interactions used to construct our algorithm. However, at least one report published since our initial analyses has now shown that GATA3 mediates genomic ESR1-binding upstream of FOXA1 [35]. This confirms that the integration of genomic and PPI data by our method has the capacity to identify new and otherwise unanticipated relationships with biologic significance.

### Enhanced network specificity provided by logit-lapnet

Another advantageous feature of Logit-Lapnet is the potential functional specificity of the subnetworks delineated by our algorithm. Subnetworks identified by Logit-Lapnet allow investigators to more readily focus on key genes for subsequent downstream functional analyses. For example, the subnetwork connecting AR, ESR1 and RARA identified by our algorithm in TNBC represents a cluster of genes involved in hormone receptor signaling (Figure 2). The subnetwork 2 identified by our method contains the genes PLK1, BCL2, BNIPL, and tumor suppressor p53 binding protein TP53BP2. PLK1, BNIPL and BCL2 are well-known oncogenes and part of TP53 pathway [36]. The expression of BCL2 has been proposed as a prognostic marker for breast cancer patients. This subnetwork is also interesting, as it predicts a relationships between gene products that have been previously shown to impact the G1-S (TP53BP) and G2-M cell cycle checkpoints (PLK1). Dysregulation of both G1-S and G2-M are key hallmarks of human cancer as defined by Weinberg and others. Furthermore, TP53 dysfunction has been previously shown to lead to the overexpression of PLK1 and other gene products important for driving cells with genomic instability through the cell cycle. Thus, the ability of Logit-Lapnet to detect this relationship underscores its capacity to detect key events in breast and other human cancers. These results convey a clear message that interactions between genes in clusters might be biologically relevant.

In contrast, alternative algorithms such as elastic net identify fewer subnetworks containing larger number of genes. This means that subnetworks identified by Logit-Lapnet allow investigators to more readily focus on key targets for subsequent downstream functional validation. Although network modularization methods can be used to further dissemble networks defined by elastic net into smaller modules, these smaller modules may not reflect direct interactions between their individual components. This is because elastic net defines groups of co-expressed genes rather than networks of functionally interacting gene products. Alternatively, use of Logit-Lapnet can be considered to place gene correlations in the context of biologic function. Thus, its use can be reasonably anticipated to define relationships that are more likely to be biologically and clinically relevant.

In summary, the proposed algorithm identified set of genes associated with breast tumors. In addition, integration of PPI in the algorithm enables us to recover the genes not only in association to the breast cancer subtypes but also their interacting partners which are also breast cancer related. Most importantly, from the comparison with elastic net and lasso, our method selected a reasonable size of genes and is the only algorithm capable of identified all three marker genes in defining TNBC.

## Conclusion

We have developed a graph Laplacian regularized logistic regression model for selecting the genes or network modules associated with clinical variables from gene expression profiles. Through simulation studies, we have demonstrated that our approach is much more sensitive for identifying clinically relevant genes and network modules. We have presented a case study on network module identification for triple negative breast cancer sub-type using mRNA-seq data. Our results indicate that Logit-Lapnet is a superior algorithm compared to Lasso and elastic net in disease gene and network module selection. Further work to biologically validate our predicted modules is needed to gain a more complete picture of the regulatory process in the TNBC sub-type. Beyond genomics, there are many potential applications of Logit-Lapnet to utilize the rich information provided by network structure, such as metabolomics and proeteomics studies. In conclusion, our work developing the network structure regularized logistic regression model has many implications and has provided a new tool for research in genomic studies.

## Competing interests

The authors declare that they have no competing interests.

## Authors' contributions

Genevera Allen and Zhandong Liu developed the model and wrote the paper. Wen Zhang, Ying-Wooi Wan and Kaifang Pang collected the real application data and conducted the application. Matthew Anderson provided biologic interpretation of networks and assisted with writing the paper. Zhandong Liu designed the simulation and real applications. Wen Zhang and Zhandong Liu realize and analyze the simulations.

## Supplementary Material

Additional file 1 **Proofs of lemma and theorem**. This file includes the mathematics proofs on lemma 1, 2 and theorem 1. Click here for file

Additional file 2 **Genes identified by elastic net**. This file includes a figure of genes and their respective subnetworks of PPI identified by elastic net. Genes with larger font indicates its association to breast cancer reported in the literature. Click here for file

## Declarations

The publication fee is supported by the Houston Endowment.

This article has been published as part of *BMC Genomics *Volume 14 Supplement 8, 2013: Selected articles from the International Conference on Intelligent Biology and Medicine (ICIBM 2013): Genomics. The full contents of the supplement are available online at http://www.biomedcentral.com/bmcgenomics/supplements/14/S8.
