# Supplementary material for: Molecular pathway identification using biological network-regularized logistic models
Source: BMC Genomics. 2013 Dec 9;14(Suppl 8):S7. doi: 10.1186/1471-2164-14-S8-S7 (PMC4046566; doi:10.1186/1471-2164-14-S8-S7)
Supplement: Additional file 1 — Proofs of lemma and theorem. This file includes the mathematics proofs on lemma 1, 2 and theorem 1. [file 1471-2164-14-S8-S7-S1.PDF]

Proof of Lemma 1

Proof. For fixed $\lambda > 0$, $0 < \alpha < 1$, if $\hat{\beta}_i \neq \hat{\beta}_j$, take $\hat{\beta}^*$ such that $\hat{\beta}^*_k = \hat{\beta}_k$ when $k \neq i$ and $k \neq j$. When $k = i$ or $k = j$, let $\hat{\beta}^*_k = \frac{1}{2}(\hat{\beta}_i + \hat{\beta}_j)$. Since $\mathrm{x}_i = \mathrm{x}_j$, $\mathrm{X}\hat{\beta}^* = \mathrm{X}\hat{\beta}$. As for $1 \leq i \leq n$, $X_i\hat{\beta}^* = X_i\hat{\beta}$. While the objective function $\mathbb{L}(\lambda, \alpha, \beta)$ is strictly convex, so $\mathbb{L}(\lambda, \alpha, \hat{\beta}^*) < \mathbb{L}(\lambda, \alpha, \hat{\beta})$. At the same time, $\hat{\beta}$ satisfies equation (4), which leads to a contradiction. Consequently, $\hat{\beta}_i = \hat{\beta}_j$ must hold. If $\hat{\beta}_i\ \hat{\beta}_j < 0$, take the same $\hat{\beta}^*$ again. According to the triangle inequality, $|\hat{\beta}^*|_1 < |\hat{\beta}|_1$, meaning that $\hat{\beta}$ is not a solution for lasso problem, which is also a contradiction. Thus $\hat{\beta}_i\ \hat{\beta}_j \geq 0$ holds. Also, $X_i\hat{\beta}^* = X_i\hat{\beta}$, $1 \leq i \leq n$, for $\hat{\beta}^*$ defined in the condition. So $\hat{\beta}^*$ is also minimizer of $\mathbb{L}(\lambda, \alpha, \beta)$. ■

Proof of Theorem 1

Proof. Since $\hat{\beta}_i(\lambda, \alpha)\ \hat{\beta}_j(\lambda, \alpha) > 0$, both $\hat{\beta}_i$ and $\hat{\beta}_j$ are non-zeros and $\mathrm{sign}(\hat{\beta}_i) = \mathrm{sign}(\hat{\beta}_j)$, because $\hat{\beta} = \arg\min_\beta \mathbb{L}(\lambda, \alpha, \beta)$, $\hat{\beta}$ satisfies $\frac{\partial \mathbb{L}}{\partial \beta_k}|_{\beta=\hat{\beta}} = 0$ if $\hat{\beta}_k(\lambda, \alpha) \neq 0$. Hence,

$$(-\mathrm{y} + \frac{e^{\mathrm{X}\hat{\beta}}}{1 + e^{\mathrm{X}\hat{\beta}}})^T \mathrm{x}_k + \lambda\alpha\ \mathrm{sign}(\hat{\beta}_k) + 2\lambda(1-\alpha)\hat{\beta}^T L_k = 0, \tag{8}$$

where L_k is the kth column of the Laplacian matrix L. The reduction of left hand side of equation (8) is given in the next subsection. Hence

$$(-\mathrm{y} + \frac{e^{\mathrm{X}\hat{\beta}}}{1 + e^{\mathrm{X}\hat{\beta}}})^T \mathrm{x}_i + \lambda\alpha\ \mathrm{sign}(\hat{\beta}_i) + 2\lambda(1-\alpha)\hat{\beta}^T L_i = 0 \tag{9}$$

$$(-\mathrm{y} + \frac{e^{\mathrm{X}\hat{\beta}}}{1 + e^{\mathrm{X}\hat{\beta}}})^T \mathrm{x}_j + \lambda\alpha\ \mathrm{sign}(\hat{\beta}_j) + 2\lambda(1-\alpha)\hat{\beta}^T L_j = 0 \tag{10}$$

Subtracting (10) from (9), we get

$$(-\mathrm{y} + \frac{e^{\mathrm{X}\hat{\beta}}}{1 + e^{\mathrm{X}\hat{\beta}}})^T (\mathrm{x}_i - \mathrm{x}_j) + 2\lambda(1-\alpha)\hat{\beta}^T (L_i - L_j) = 0 \tag{11}$$

According to property of the Laplacian matrix L,

$$\hat{\beta}^T (L_i - L_j) = \hat{\beta}_i - \hat{\beta}_j \tag{12}$$

From (11), (12) along with Cauchy–Schwartz inequality and the property of L_1 norm, we know

$$|\hat{\beta}_i - \hat{\beta}_j| \leq \frac{1}{2\lambda(1-\alpha)} |\mathrm{y} - \frac{e^{\mathrm{X}\hat{\beta}}}{1 + e^{\mathrm{X}\hat{\beta}}}|_1 \cdot |\mathrm{x}_i - \mathrm{x}_j|_1 \tag{13}$$

Since $\hat{\beta}$ is the minimizer, the residual $|\mathrm{y} - \frac{e^{\mathrm{X}\hat{\beta}}}{1+e^{\mathrm{X}\hat{\beta}}}|_1 < |\mathrm{y} - \frac{e^{\mathrm{X}\beta}}{1+e^{\mathrm{X}\beta}}|_1 \leq |\mathrm{y}|_1 + |\frac{e^{\mathrm{X}\beta}}{1+e^{\mathrm{X}\beta}}|_1$ which tends to $|\mathrm{y}|_1$ as β tends to negative infinity. So

$$|\hat{\beta}_i - \hat{\beta}_j| < \frac{1}{2\lambda(1-\alpha)} |\mathrm{y}|_1 \cdot |\mathrm{x}_i - \mathrm{x}_j|_1 \tag{14}$$

Since $\mathbf{X}$ are standardised, $|x_i - x_j|_1 = \sqrt{2(1-\rho)}$, dividing both sides of (14) by $|\mathrm{y}|_1$ leads to equation (5). ■

Proof of Lemma 2

Proof. Taking the partial derivative of $l(\lambda, \alpha, \beta)$ with respect to β, we get

$$\frac{\partial l}{\partial \beta} = -\mathrm{X}^T\mathrm{y} + \mathrm{X}^T \cdot \frac{e^{\mathrm{X}\beta}}{1+e^{\mathrm{X}\beta}} + \lambda\alpha \ \mathrm{sign}(\beta) + 2\lambda(1-\alpha)L\beta. \tag{15}$$

In (15), the sign of the divisor denotes element-wise division. From KKT conditions, we know if $\beta = 0$, λ will make $\frac{\partial l}{\partial \beta}$ zero. That is,

$$\frac{\partial l}{\partial \beta}|_{\beta=0} = -\mathrm{X}^T\mathrm{y} + \frac{1}{2}\mathrm{X}^T \cdot \mathbf{1}_n + \lambda\alpha \ \mathbf{1}_p = \mathbf{0}_p, \tag{16}$$

where $\mathbf{0}_p$ is a column vector which contains p zero elements. Hence,

$$\lambda\alpha \ \mathbf{1}_p = \mathrm{X}^T\mathrm{y} - \frac{1}{2}\mathrm{X}^T \cdot \mathbf{1}_n. \tag{17}$$

The dimensions of $\mathbf{1}_p$ and $\mathbf{1}_n$ here are $p \times 1$ and $n \times 1$ respectively. Taking L_∞ norm on both sides of (17) leads to

$$\alpha\lambda = |\mathrm{X}^T\mathrm{y} - \frac{1}{2}\mathrm{X}^T \cdot \mathbf{1}_n|_\infty \leq |\mathrm{X}^T\mathrm{y}|_\infty + \frac{1}{2}|\mathrm{X}^T \cdot \mathbf{1}_n|_\infty. \tag{18}$$

Thus

$$\lambda \leq \frac{|\mathrm{X}^T\mathrm{y}|_\infty + \frac{1}{2}|\mathrm{X}^T \cdot \mathbf{1}_n|_\infty}{\alpha} \tag{19}$$

and we can take

$$\lambda_{\max} = \frac{2|\mathrm{X}^T\mathrm{y}|_\infty + |\sum_{i=1}^n X_i^T|_\infty}{2\alpha} \tag{20}$$

which is the condition in Lemma 3. ■
