# Supplementary figures and images for: Molecular pathway identification using biological network-regularized logistic models
Source: BMC Genomics. 2013 Dec 9;14(Suppl 8):S7. doi: 10.1186/1471-2164-14-S8-S7 (PMC4046566; doi:10.1186/1471-2164-14-S8-S7)

# Elastic Net

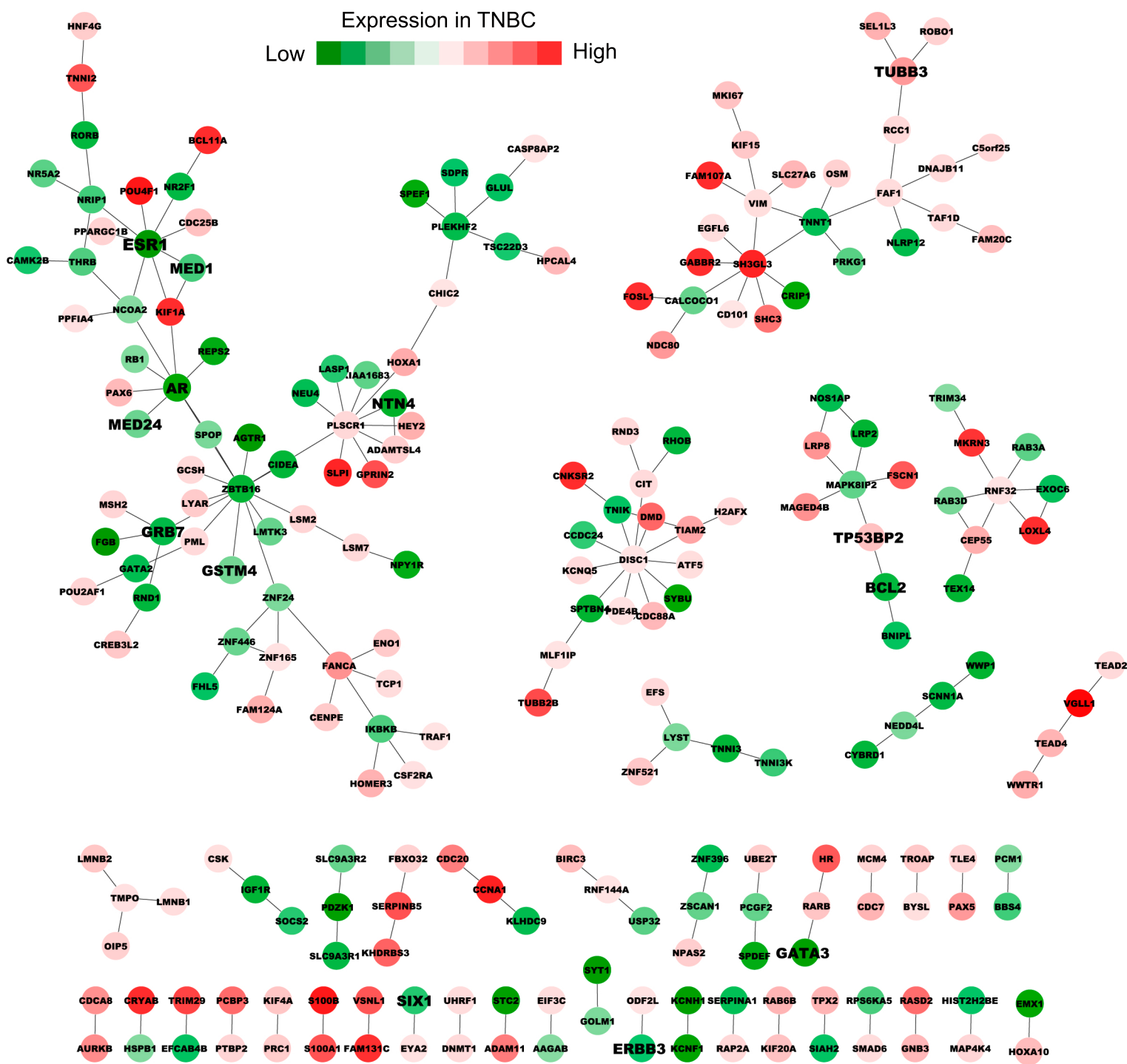

Supplement: Additional file 2 — Genes identified by elastic net. This file includes a figure of genes and their respective subnetworks of PPI identified by elastic net. Genes with larger font indicates its association to breast cancer reported in the literature. [file 1471-2164-14-S8-S7-S2.PDF]
